# Synthesis, Crystal Structure, and Optical and Magnetic Properties of the New Quaternary Erbium Telluride EuErCuTe_3_: Experiment and Calculation

**DOI:** 10.3390/ma17102284

**Published:** 2024-05-11

**Authors:** Anna V. Ruseikina, Maxim V. Grigoriev, Ralf J. C. Locke, Vladimir A. Chernyshev, Alexander A. Garmonov, Thomas Schleid

**Affiliations:** 1 Laboratory of Theory and Optimization of Chemical and Technological Processes, University of Tyumen, Tyumen 625003, Russia; maxgrigmvv@ya.ru; 2 Institute for Inorganic Chemistry, University of Stuttgart, D-70569 Stuttgart, Germany; 3 Institute of Natural Sciences and Mathematics, Ural Federal University Named after the First President of Russia B.N. Yeltsin, Ekaterinburg 620002, Russia; vchern@inbox.ru; 4 Institute of Physics and Technology, University of Tyumen, Tyumen 625003, Russia; gamma125@mail.ru

**Keywords:** quaternary erbium telluride, synthesis, crystal structure, magnetic measurements, DFT calculations

## Abstract

This paper reports for the first time on a new layered magnetic heterometallic erbium telluride EuErCuTe_3_. Single crystals of the compound were obtained from the elements at 1120 K using CsI as a flux. The crystal structure of EuErCuTe_3_ was solved in the space group *Cmcm* (*a* = 4.3086(3) Å, *b* = 14.3093(9) Å, and *c* = 11.1957(7) Å) with the KZrCuS_3_ structure type. In the orthorhombic structure of erbium telluride, distorted octahedra ([ErTe_6_]^9−^) form two-dimensional layers (Er(Te1)2/2e(Te2)4/2k−)∞2, while distorted tetrahedra ([CuTe_4_]^7−^) form one-dimensionally connected substructures (Cu(Te1)2/2e(Te2)2/1t5−∞1) along the [100] direction. The distorted octahedra and tetrahedra form parallel two-dimensional layers (CuErTe32−∞2) between which Eu^2+^ ions are located in a trigonal-prismatic coordination environment (${\left[{EuTe}_{6}\right]}^{10-})$. The trigonal prisms are connected by faces, forming chains (Eu(Te1)2/2(Te2)4/22−∞1) along the [100] direction. Regularities in the variations in structural parameters were established in the series of erbium chalcogenides (EuErCu*Ch*_3_ with *Ch* = S, Se, and Te) and tellurides (Eu*Ln*CuTe_3_ with *Ln* = Gd, Er, and Lu). Ab-initio calculations of the crystal structure, phonon spectrum, and elastic properties of the compound EuErCuTe_3_ were performed. The types and wavenumbers of fundamental modes were determined, and the involvement of ions in the IR and Raman modes was assessed. The experimental Raman spectra were interpreted. The telluride EuErCuTe_3_ at temperatures below 4.2 K was ferrimagnetic, as were the sulfide and selenide derivatives (EuErCu*Ch*_3_ with *Ch* = S and Se). Its experimental magnetic characteristics were close to the calculated ones. The decrease in the magnetic phase transition temperature in the series of the erbium chalcogenides was discovered.

## 1. Introduction

Heterometallic chalcogenides based on erbium have been of constant interest to researchers due to their structural possibilities, including their channel, tunnel, and layered structures, as well as their potential valuable optoelectronic, magnetic, semiconductor, and thermoelectric properties [1,2,3,4,5,6,7,8,9,10,11,12,13]. Doping erbium chalcogenides improves the optical characteristics of the material in terms of conductivity, reduces the bandgap energy, and enhances electrical conductivity and light absorption. Erbium-doped tellurides have found potential applications in solar cell and optical devices [14]. New heterometallic erbium chalcogenides are formed in ternary systems, such as *M*_2_*Ch*–Eu*Ch*–Er_2_*Ch*_3_ (*M* = *d*-element and *Ch* = chalcogen) [15,16,17,18], and they can combine the properties of corresponding binary phases. Magnetic semiconductors based on europium chalcogenides are attractive due to their wide range of magnetic properties, as they can be ferro-, meta-, or antiferromagnetic [19]. They exhibit high saturation magnetization, a strong magneto-optical effect, magnetoresistance, and spin filtration effects, all arising from the unusual combination of electronic, magnetic, and optical properties in the europium chalcogenides [19,20,21]. Europium chalcogenides have the potential to create new magnetoelectronic devices, including magnetic random-access memory and magnetic tunneling transistors [19,20]. Bulk EuTe is an antiferromagnetic Heisenberg II type with a Néel temperature of *T*_N_ = 9.6 K [21,22,23,24] and a wide bandgap semiconductor with a bandgap width of 2.5 eV [24]. The optical and magnetic properties of europium tellurides can be controlled by changing their sizes [21,24,25]. In isolated monolayers, both ferrimagnetic and antiferromagnetic phase transitions can occur [23], and nanoparticles exhibit pronounced superantiferromagnetic transitions between 2 K and 20 K [21].

The compounds EuErCu*Ch*_3_ (*Ch* = S, Se, and Te) contain two magnetically active cations, Eu^2+^ and Er^3+^, which contribute to the magnetic ordering of the chalcogenides. The magnetic moment of the Er^3+^ ion (9.59 μ_B_) is larger than that of the Eu^2+^ ion (7.94 μ_B_), suggesting that the EuErCu*Ch*_3_ compounds exhibit ferrimagnetic properties [16,18]. Indeed, the EuErCu*Ch*_3_ compounds (*Ch* = S and Se) undergo a transition from ferrimagnetic to paramagnetic states at 4.8 K [18] and 4.7 K [16], respectively. The synthesis and properties of EuErCuTe_3_ have not been reported yet. However, based on the magnetic ions involved, a ferrimagnetic transition can be expected in this compound. The orthorhombic EuErCu*Ch*_3_ compounds (*Ch* = S and Se) crystallize in the space group *Pnma* (a structural type Eu_2_CuS_3_) and the space group *Cmcm* (a structural type KZrCuS_3_), respectively. Thus, with an increase in the chalcogen radius from *r_i_*(S^2−^) = 1.84 Å to *r_i_*(Se^2−^) = 1.98 Å [26], a change in the space group to a higher symmetry can occur. According to L.A. Koscielski's review [17], compounds of this type crystallize only in the *Pnma* and *Cmcm* space groups, and so it can be assumed that despite a further increase in the chalcogen radius to *r_i_*(Te^2−^) = 2.21 Å [26], the EuErCuTe_3_ compound will crystallize in the *Cmcm* space group. Quantum-mechanical calculations have been previously performed for the EuErCuTe_3_ compound, assuming it crystallizes in the KZrCuSe_3_ structural type [27,28].

Recently, the chalcogenidation of a multi-component oxide mixture obtained by the thermolysis of co-crystallized nitrates [3,29,30,31] or commercial oxides [31,32] has been actively used for the synthesis of quaternary chalcogenides, providing a high yield of the target phase. However, the toxicity and instability of tellurium hydride limit the applicability of this method for obtaining EuErCuTe_3_. In our opinion, the most effective and safe method for obtaining this compound would be the halide flux method using elements, which allows for the growth of single crystals in a sealed ampoule, with a relatively low synthesis temperature, and the absence of impurities would enable the study of the physical properties of the samples.

The aim of this study was to synthesize single crystals of the quaternary telluride EuErCuTe_3_, determine its crystal structure, and investigate its magnetic and optical properties. In addition, computational studies were conducted to shed light on the optical properties of EuErCuTe_3_.

## 2. Experimental

### 2.1. Materials

Eu (99.99%), Er (99.9%), CsI (99.9%), and Te (99.9%) were purchased from ChemPur (Karlsruhe, Germany). Cu (99.999%) was obtained from Aldrich (Milwaukee, WI, USA).

### 2.2. Synthesis

**Figure 1:**
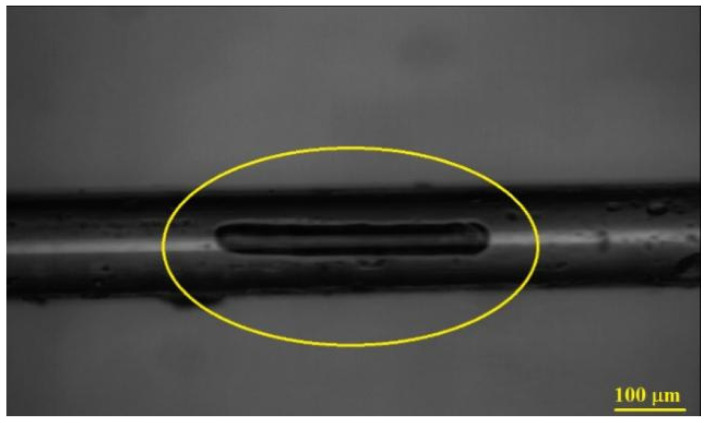
Photograph of an EuErCuTe_3_ (in yellow circle) crystal placed in a capillary for the X-ray diffraction analysis (the single crystal image was made using a Horiba XploRA Raman spectrometer (HORIBA Scientific, Kyoto, Japan)).

Single crystals of EuErCuTe_3_ were synthesized by mixing stoichiometric amounts of copper, tellurium, and lanthanide elements (1 Cu: 1 Eu: 1 Er: 3 Te) in the presence of an excess of CsI halide flux in a glovebox under an argon atmosphere. The weighing of the samples into quartz ampoules was carried out in an inert atmosphere inside the glovebox. The glovebox was used to prevent the interaction of the elemental substances with the oxygen and carbon dioxide in the air and water vapors, which would lead to the formation of thermodynamically stable oxides, carbonates, and hydroxycarbonates at room temperature [33,34]. To prevent the formation of silicate oxides during the synthesis process due to the interactions of the starting components with quartz, a pyrolytic thin layer of amorphous carbon was applied to the inner wall of the quartz ampoules prior to beginning. The quartz ampoules were evacuated to a pressure of 2 × 10^−3^ mbar, sealed, and heated in a muffle furnace from room temperature to 1120 K. The heating was carried out from room temperature to 1120 K for 30 h and kept at this temperature for 96 h, then it was cooled to 570 K for 140 h, and finally, it was cooled to room temperature for 3 h. The reaction product was purified from the residual flux using demineralized water. The product consisted of black needle-like crystals of EuErCuTe_3_ with sizes of up to 500 μm (Figure 1). The obtained crystals were suitable for single crystal X-ray diffraction analysis and taking measurements of their magnetic and optical properties. Unfortunately, high-quality powder diffraction patterns could not be obtained, as copper compounds are strong absorbers of molybdenum radiation.

### 2.3. X-ray Diffraction Analysis

The intensities from a single crystal of the EuErCuTe_3_ of 0.05 × 0.05 × 0.45 mm^3^ dimensions were collected at 293(2) K using a SMART APEX II single-crystal diffractometer (Bruker AXS, Billerica, MA, USA) equipped with a CCD-detector, graphite monochromator, and Mo-*K*_α_ radiation source. The parameters of the elementary cell were determined and refined for a set of 11880 reflections. The parameters of the elementary cell corresponded to the orthorhombic crystal system. The space group *Cmcm* was determined from the statistical analysis of all the intensities. Absorption corrections were applied using the SADABS (2008) program. The crystal structure was solved by direct methods using the SHELXS (2013) program and refined in an anisotropic approximation using the SHELXL (2013) program [35]. Structural investigations for the presence of missing symmetry elements were conducted using the PLATON (2009) program [36]. The crystallographic data were deposited in the Cambridge Crystallographic Data Centre. The data can be downloaded from www.ccdc.cam.ac.uk/data_request/cif (accessed on 25 February 2024).

### 2.4. Electron-Beam Microprobe Analysis

The SEM (scanning electron microscopy) image of the EuErCuTe_3_ was acquired using an electron-beam X-ray microprobe (SX-100, Cameca, Gennevilliers, France). The EDX (energy dispersive X-ray spectroscopy) spectra for several examples roughly confirmed the 1:1:1:3 stoichiometry of all the investigated EuErCuTe_3_ compounds.

### 2.5. Magnetic Measurements

Magnetic measurements were performed with a Quantum Design Magnetic Property Measurement System (MPMS3), San Diego, CA, USA. The SOUID-magnetometer was used to measure the temperature dependence of the EuErCuTe_3_ sample’s magnetic moment under a magnetic field of 40 kA·m^−1^. These measurements took place in the temperature range 2 to 300 K using the zero-field cooling (ZFC) and field cooling (FC) modes. The isothermal magnetization of up to 4 MA·m^−1^ was measured at 2 K and 300 K.

### 2.6. Spectroscopy of the Raman Scattering

Raman spectra of the single crystal sample of EuErCuTe_3_ were acquired using a Horiba XploRa spectrometer (HORIBA Scientific, Kyoto, Japan). The excitation light at a wavelength of 532 nm was used. The acquisition conditions were as follows: filter—10, hole—300, slit—100, and resolution—2400.

### 2.7. DFT Calculations

Calculations were performed at the theoretical level DFT (density functional theory)/B3LYP. In this approach, we took into account the nonlocality of the exchange interaction, which was necessary for describing the compounds with ionic and covalent bonds. The calculations were carried out in the program CRYSTAL17 [37]. For the rare-earth ions, quasi-relativistic pseudopotentials with attached basis sets were used. We used the pseudopotentials ECP53MWB and ECP57MWB with attached basis sets of the TZVP type for the outer shells (5*s*^2^5*p*^6^) [38]. The all-electron basis set “Cu_86-4111(41D)G_doll_2000” was used for the copper [37]. For the tellurium, an all-electron basis was also used [39]. Any diffuse functions with exponents smaller than 0.1 were deleted from the basis sets. A self-consistent field was calculated with an accuracy of 10^−9^ a.u. The Monkhorst-Pack mesh used was 8 × 8 × 8.

The use of the pseudopotential for the inner shells of a rare-earth ion reduced the cost of the computer resources and allowed us to calculate the structure and the dynamics of the crystal lattice adequately [15].

The elastic constants and phonon spectrum were calculated for the previously optimized crystal structure.

## 3. Results

### 3.1. Crystal Structures of the EuErCuTe_3_

According to the X-ray crystallographic analysis of the single crystals, the compound EuErCuTe_3_ crystallized in the orthorhombic space group *Cmcm* with a KZrCuS_3_ structural type. The crystallographic data, data collection details, atomic coordinates, thermal displacement parameters, bond lengths, and valence angles are presented in Table 1, Table 2, Table 3 and Table 4. A similar structural type was observed in the erbium quarter selenide compound EuErCuSe_3_ [16,40]. The lattice constants obtained from the DFT calculations *a* = 4.3401 Å, *b* = 14.2459 Å, and *c* = 11.2309 Å were in good agreement with those determined experimentally (Table 1).

**Figure 2:**
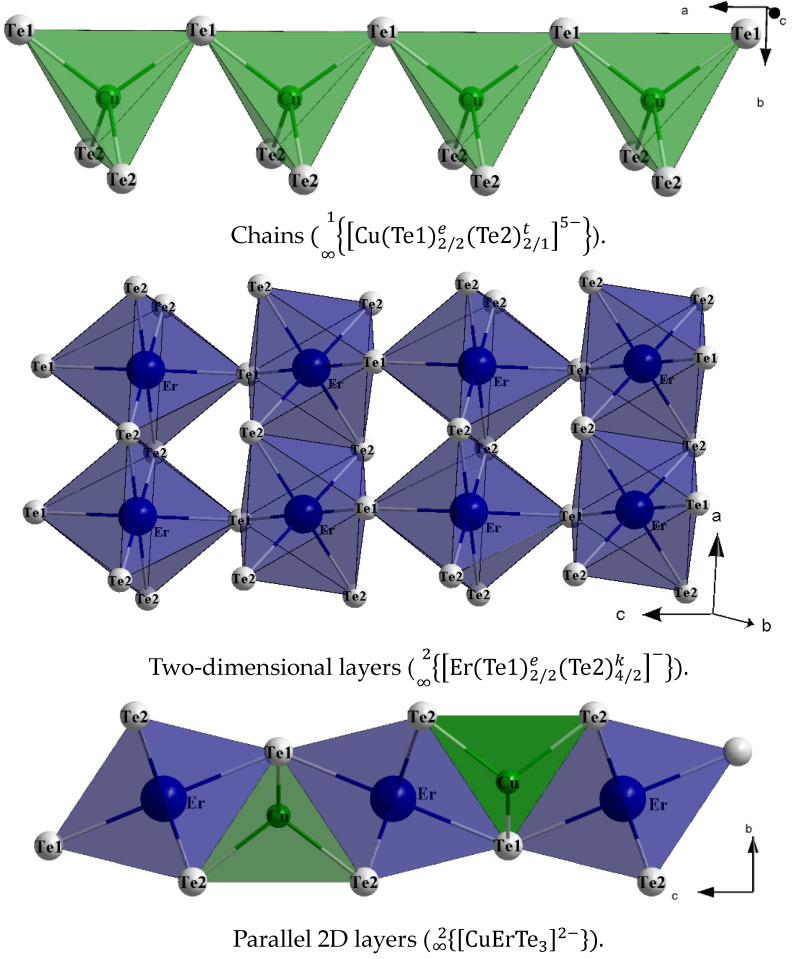

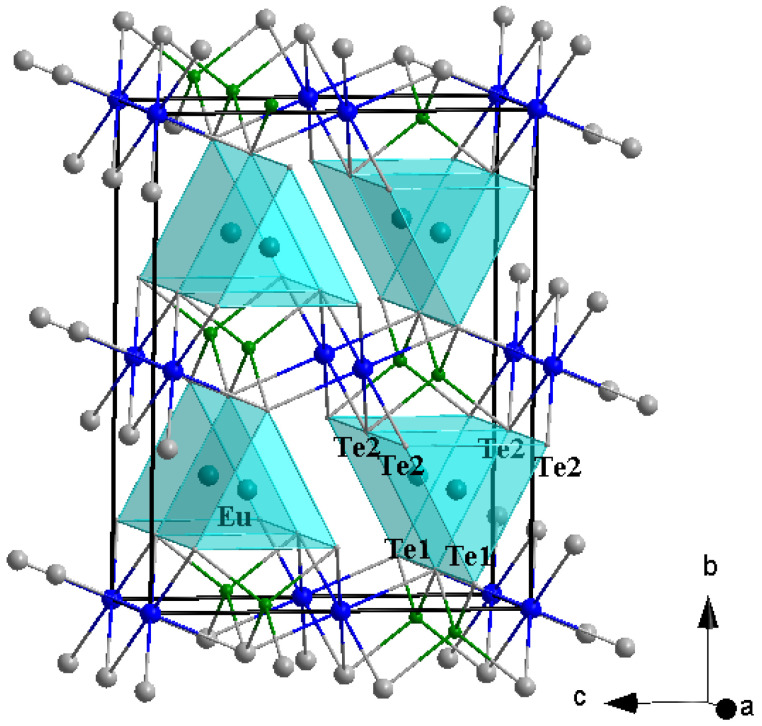
Crystal structure of the EuErCuTe_3_.

The crystal structure of EuErCuTe_3_ has a layered-block structure (Figure 2).

The cations Eu^2+^, Er^3+^, and Cu^+^ occupy independent crystallographic positions. In the EuErCuTe_3_ compound, the structure was formed by distorted tetrahedra (${\left[{CuTe}_{4}\right]}^{7–}$), octahedra (${\left[{ErTe}_{6}\right]}^{9–})$, and trigonal prisms (${\left[{EuTe}_{6}\right]}^{10–})$. The sums of the valence forces for the EuErCuTe_3_ compound, taking into account coordination, were Eu (1.64), Er (2.99), and Cu (1.42).

Distorted tetrahedra form chains (Cu(Te1)2/2e(Te2)2/1t5–∞1) along the [100] direction through shared vertex atoms (Te1) (Figure 2). In the tetrahedra, the distances *d*(Cu–Te) are equal to 2.641(1) Å and 2.667(1) Å (Table 4), indicating a deviation from the theoretical value of *d*(Cu–Te) = 2.81 Å (calculated based on *r_i_*(Cu^+^) = 0.6 Å, coordination number (*C.N.*) = 4; *r_i_*(Te^2−^) = 2.21 Å) [26]), which is associated with an increase in the covalent component of the chemical bond.

The values of the valence angles ∠(Te–Cu–Te) deviated from the value of the ideal tetrahedral angle (Table 4). The distortions of the tetrahedra in the EuErCuTe_3_ structure were evaluated using the τ_4_ descriptor [41]. The value of τ_4_ was 0.978, indicating a distortion in the coordination geometry around the Cu^+^ by 15% from an ideal tetrahedral structure to a trigonal-pyramidal structure. The degree of distortion in the tetrahedral polyhedra in the telluride EuErCuTe_3_ was higher than that in the selenide EuErCuSe_3_ [16] and sulfide EuErCuS_3_ [15], for which the values of τ_4_ were 0.984 and 0.986, and the distortion indexes were 11% and 9%, respectively.

Between the chains of tetrahedra, there were distorted octahedra ([ErTe_6_]^9−^) with bond lengths of 3.0277(4) Å and 3.0423(5) Å (Table 4) compared to the theoretical value of *d*(Er–Te) = 3.1 Å (*r_i_*(Er^3+^) = 0.89 Å, *C.N.* = 4) [26]. The values of the valence angles ∠(Te–Er–Te) deviated from the value of the ideal octahedral angle (Table 4). The distorted octahedra formed two-dimensional layers (Er(Te1)2/2e(Te2)4/2k−∞2) through the shared vertex atoms (Te1) along the [001] direction and the shared edges (Te2Te2). The distorted octahedra and tetrahedra formed parallel two-dimensional layers (CuErTe32−)∞2 in the [101] plane. Between these layers, there were trigonal prisms that were connected by the faces Te2Te2Te1, forming chains (Eu(Te1)2/2(Te2)4/22−∞1) along the [100] direction. The theoretical value of *d*(Eu–Te) is 3.38 Å (*r_i_*(Eu^2+^) = 0.89 Å, *C.N*. = 4) [26]. In the EuErCuTe_3_ structure, six Eu–Te distances were shorter than 3.35 Å, while the seventh and eighth distances were larger than the theoretical value and measured 3.86 Å, which was not accounted for in the coordination polyhedron due to weak interactions.

Thus, the three-dimensional crystal structure of EuErCuTe_3_ was formed by two-dimensional layers consisting of octahedra and tetrahedra in the *bc* plane separated by one-dimensional chains of trigonal prisms.

In the series of erbium chalcogenides (EuErCu*Ch*_3_ (*Ch* = S, Se, and Te)), a change in the space group from *Pnma* (EuErCuS_3_) to *Cmcm* (EuErCuSe_3_ and EuErCuTe_3_) and a change in structural type from Eu_2_CuS_3_ to KZrCuS_3_, respectively, were observed. As the chalcogen radius increased in the compounds (EuErCu*Ch*_3_ (*Ch* = S [15], Se [16], and Te (Table 1))), an increase in the unit cell parameters was observed, as follows:

*a_Pnma_*(*c_Cmcm_*) = 10.1005(2) Å ($a_{{EuErCuS}_{3}}$) → 10.4602(7) Å ($c_{{EuErCuSe}_{3}}$)→ 11.1957(7) Å ($c_{{EuErCuTe}_{3}}$);
*b_Pnma_*(*a_Cmcm_*) = 3.91255(4) Å ($a_{{EuErCuS}_{3}}$) → 4.0555(3) Å ($c_{{EuErCuSe}_{3}}$)→ 4.3086(3) Å ($c_{{EuErCuTe}_{3}}$);
*c_Pnma_*(*b_Cmcm_*) = 12.8480(2) Å ($a_{{EuErCuS}_{3}}$) → 13.3570(9) Å ($c_{{EuErCuSe}_{3}}$)→ 14.3093(9) Å ($c_{{EuErCuTe}_{3}}$).

Correspondingly, the volume of the unit cell increased as follows: 507.737(14) Å^3^ (EuErCuS_3_) [15] → 566.62(6) Å^3^ (EuErCuSe_3_) [16] → 690.25(8) Å^3^ (EuErCuTe_3_) (Table 1).

A regular increase in the average metal-chalcogen bond lengths was also observed in the chalcogenide series. For example, we observed the following:

*d*(Eu–*Ch*): 3.060 Å (*Ch* = S) → 3.130 Å (*Ch* = Se) → 3.330 Å (*Ch* = Te);
*d*(Er–*Ch*): 2.723 Å (*Ch* = S) → 2. 730 Å (*Ch* = Se) → 3.037 Å (*Ch* = Te);
*d*(Cu–*Ch*): 2.350 Å (*Ch* = S) → 2.468 Å (*Ch* = Se) → 2.654 Å (*Ch* = Te).

Thus, the increase in the chalcogen radius led to the transformation of the local geometry around the Eu^2+^, resulting in a change in the type of its coordination polyhedron from a one-capped trigonal prism in EuErCuS_3_ [15] to a trigonal prism in EuErCuSe_3_ and EuErCuTe_3_, a change in the structural type and space group in the series of EuErCu*Ch*_3_ compounds, and an increase in structural parameters.

When comparing the structural parameters of the EuErCuTe_3_ compound with the already known europium tellurides EuGdCuTe_3_ and EuLuCuTe_3_ [42], their decrease was observed with a decrease in the ionic radius of *Ln*^3+^ (*r_i_*(Gd^3+^) = 0.938 Å > *r_i_*(Er^3+^) = 0.89 Å > *r_i_*(Lu^3+^) = 0.861 Å) [26]. The unit cell parameters of Eu*Ln*CuTe_3_ (*Ln* = Gd [42], Er (Table 1), Lu) [42] changed as follows:

*a_Pnma_*(*c_Cmcm_*) = 11.3761(7) Å ($a_{{EuGdCuTe}_{3}}$) → 11.1957(7) Å ($c_{{EuErCuTe}_{3}}$) → 11.1174(7) Å ($c_{{EuLuCuTe}_{3}}$);
*b_Pnma_*(*a_Cmcm_*) = 4.3405(3) Å ($a_{{EuGdCuTe}_{3}}$) → 4.3086(3) Å ($c_{{EuErCuTe}_{3}}$)→ 4.2937(3) Å ($c_{{EuLuCuTe}_{3}}$);
*c_Pnma_*(*b_Cmcm_*) = 14.3469(9) Å ($a_{{EuGdCuTe}_{3}}$) → 14.3093(9) Å ($c_{{EuErCuTe}_{3}}$)→ 14.2876(9) Å ($c_{{EuLuCuTe}_{3}}$).

The volume of the unit cell decreased from 708.42(8) to 682.02(8) Å^3^ in the Eu*Ln*CuTe_3_ (*Ln* = Gd, Er, and Lu). Additionally, a consistent decrease in the average metal-tellurium bond length was observed in this series. Thus, we observed the following:

*d*(Eu–Te): 3.377Å → 3.330 Å → 3.332 Å;
*d*(*Ln*–Te): 3.081 Å → 3.037 Å → 3.017 Å;
*d*(Cu–Te): 2.666 Å → 2.654 Å → 2.648 Å.

Thus, the decrease in the lanthanide radius in the series of Eu*Ln*CuTe_3_ compounds (*Ln* = Gd, Er and Lu) was accompanied by a change in the structural type and space group, as well as decreases in the structural parameters.

### 3.2. Magnetic Properties of the EuErCuTe_3_

**Figure 3:**
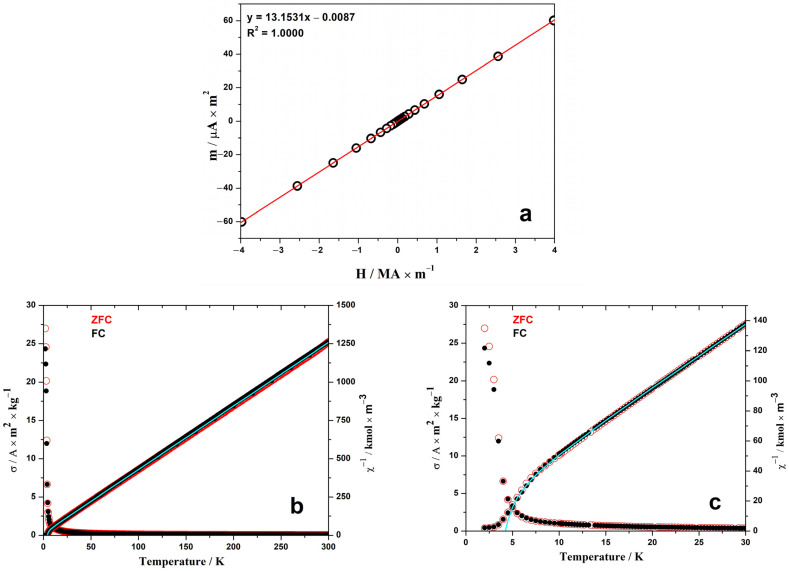
Field-dependent magnetization at 300 K (**a**) and temperature-dependent specific magnetization and reciprocal molar magnetic susceptibility at 40 MA m^−1^ (**b**,**c**) of EuErCuTe_3_ sample. The measurements’ temperature-dependent magnetizations were performed in the zero-field cooled (ZFC) and nonzero-field cooled (FC) modes.

The experimental field dependence of the magnetic moment of the sample at a temperature of 300 K had a linear form, characteristic of paramagnetic materials (Figure 3a). From this dependence, assuming the validity of the Curie law *m* = *HCT*^−1^, the Curie constant *C*_300K_ = 0.232 m^3^ K·kmol^−1^ and the corresponding effective magnetic moment *μ*_300K_ = 12.14 μ_B_ were calculated.

Given the experimental data on the temperature-dependent magnetic moment of the EuErCuTe_3_ sample, the temperature-dependent inverse molar magnetic susceptibility (Figure 3b,c) was calculated. Taking as a first approximation the Curie-Weiss law (*χ*^−1^ = *C*^−1^(*T* − *θ*_p_)) at temperatures from 50 to 300 K, the Curie constant *C*_50–300K_ = 0.241 m^3^·K·kmol^−1^ and the Weiss constant (Curie paramagnetic temperature) *θ*_p_ = −4.0 K, as well as the effective magnetic moment *μ*_50–300K_ = 12.39 μ_B_, were obtained.

The paramagnetic parameters of the EuErCuTe_3_ compound corresponded well to the calculated parameters of the free ions ($\mu =\sqrt{7.94^{2}+9.58^{2}}=12.44\;$μ_B_, *C* = 0.243 m^3^·K·kmol^−1^). However, the Curie paramagnetic temperature of this sample was negative, and the temperature dependence of the inverse susceptibility at low temperatures (Figure 3b,c) had a form characteristic of ferrimagnetic compounds. Therefore, to approximate this dependence in the temperature range 5 to 300 K, the Néel formula for a two-sublattice ferrimagnet model was used (*χ*^−1^ = *T*/*C* + *χ*_0_^−1^ − *σ*/(*T* − *θ*)). The calculations showed very good agreement between the experimental points and the theoretical model (Figure 3). The best fit was obtained with the following parameter values: *C* = 0.241 m^3^·K·kmol^−1^, *χ*_0_^−1^ = 14.5 kmol·m^−3^, *σ* = 37 kmol·K·m^−1^, and *θ* = 3.1 K. Based on these data, the Néel temperature was determined at *χ*^−1^ = 0 using the following formula: *T*_c_ = (*θ* − *C*/*χ*_0_ + ((*θ* − *C*/*χ*_0_)^2^ + 4*C*(*θ*/*χ*_0_ + *σ*))^0.5^)/2 = 4.2 K. This value was close to the observed temperature at the divergence point of the magnetization curves for the FC and ZFC modes. Similar values for the phase transition points were obtained for EuErCuSe_3_ (4.7 K [16]) and EuErCuS_3_ (5.0 K [18]).

**Figure 4:**
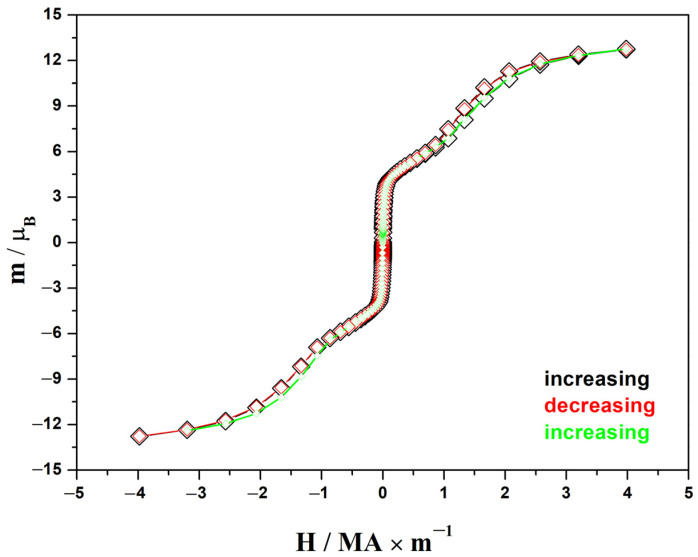
Magnetization curves of the EuErCuTe_3_ sample at 2 K.

The magnetization in Bohr magnetons per formula unit plotted at 2 K (Figure 4) confirmed the conclusion regarding the ferrimagnetic structure of the moments in this compound. The shape of this curve exhibits the metamagnetic behavior of the moments. In the external fields of 0.03 and approximately 1 MA m^−1^, jumps in susceptibility were observed, indicating a change in the magnetic structure. The saturation, which theoretically should approach the value of gS(Eu^2+^) + g_J_J(Er^3+^) = 7 μ_B_ + 9 μ_B_ = 16 μ_B_, was not achieved until reaching 4 MA·m^−1^.

The magnetic properties of the EuErCuTe_3_ compound were similar to those of EuGdCuTe_3_. Both are ferrimagnets at low temperatures (lower than 4.2 K for the first and 7.9 K for the second), unlike EuLuCuTe_3_, which is ferromagnetic under a temperature of 3.0 K.

### 3.3. Band Structure of EuErCuTe_3_

**Figure 5:**
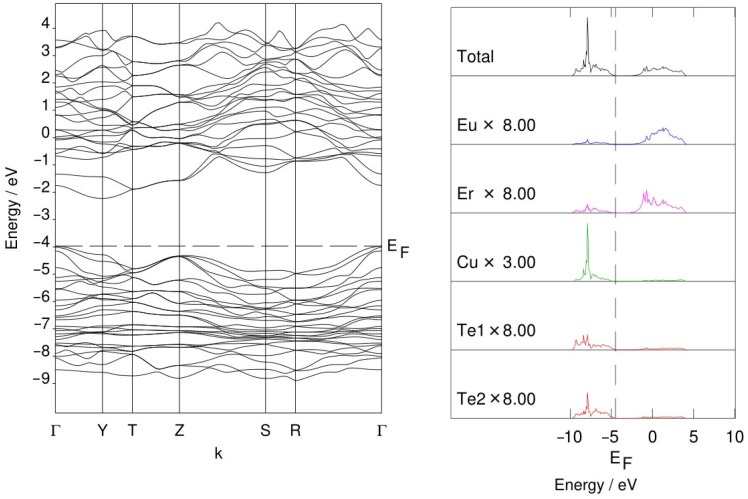
Electronic band structure of EuErCuTe_3_.

We used the points Γ(0,0,0), Y(^1^/_2_,^1^/_2_,0), T(^1^/_2_,^1^/_2_,^1^/_2_), Z(0,0,^1^/_2_), S (0,^1^/_2_,0), and R(0,^1^/_2_,^1^/_2_) at the Brillouin zone of the space group *Cmcm*. The band structure (Figure 5) did not include the 4f states of erbium and europium since they were replaced by a pseudopotential.

As can be seen from the figure, copper and tellurium orbitals are the main contributions to the states near the top of the VB. Orbitals of erbium and europium are the main contributions to the bottom of the CB. The calculations predicted for EuErCuTe_3_ an indirect band gap value of 1.75 eV (it was a HOMO–LUMO estimation). This value was close to the experimental data values for isostructural quaternary chalcogenides [16,18,43]. In the series EuErCu*Ch*_3_ (*Ch* = S, Se, and Te), decreases in the band gap widths of the compounds were observed (1.93 eV (EuErCuS_3_) [18] → 1.79 eV (EuErCuSe_3_) [16] → 1.75 eV (EuErCuTe_3_)), which was consistent with the data on the narrowing of the band gap in the chalcogenide series [44]. Isostructural europium chalcogenides have lower band gap values compared to strontium chalcogenides in the space group *Cmcm*, for example, in Sr*RE*CuSe_3_ (*RE* = Ho − Lu), the values range from 2.03–2.21 eV [43]. The narrower band gap of Eu*RE*Cu*Ch*_3_ was explained by the presence of a 4*f*–5*d* transition in the Eu^2+^ ion [45].

### 3.4. Elastic Constants and Elastic Modulus

The elastic constants and the elastic modulus of the compound EuErCuTe_3_ are presented in Table 5. This table presents the bulk module (*B*), shear module (*G*), Young’s modulus (*G*), and Poisson’s ratio. These are values for a polycrystal, and they are calculated by averaging the schemes of Voigt, Reuss, and Hill. The Voigt scheme assumes the uniformity of local strains. The Reuss scheme assumes the uniformity of local stresses. The Voigt scheme provides the upper bound, while the Reuss scheme provides the lower bound of the value. The Hill approximation provides the arithmetical average of the Voigt and Reuss values [46,47]. 

**Figure 6:**
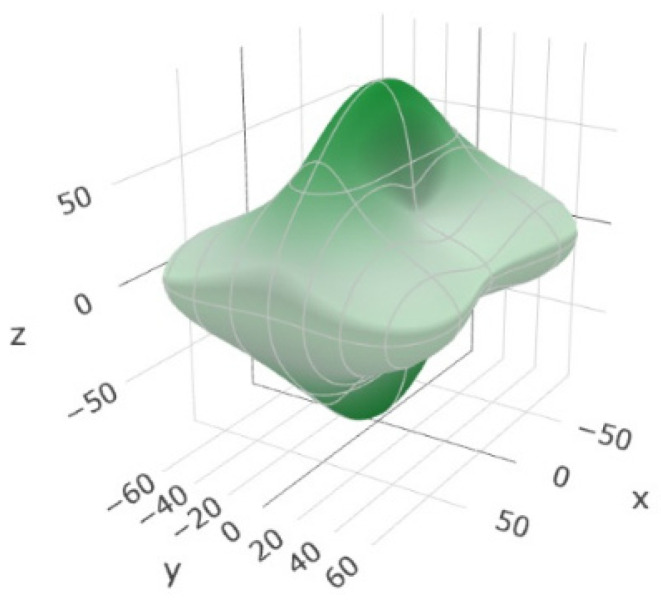
The Young’s modulus (GPa) and its dependence on direction in the crystal EuErCuTe_3_.

The Voigt and Reuss estimates were very different (Table 5), which indicated the anisotropy of the elastic properties. The dependence of the Young's modulus on direction also illustrated the strong anisotropy of the elastic properties (Figure 6).

(1) $$H_{V}=0.92{\left(\frac{G}{B}\right)}^{1.137}G^{0.708}$$

The empirical Formula (1) was used to calculate the hardness. According to [48] the formula was based on correlations between the Vickers hardness (*H_V_*) and the ratio of the shear and bulk moduli. The parameters of the formula were determined from reproducing the hardness of more than forty compounds with ionic and covalent bonds [48]. In (1), the shear (*G*) and bulk (*B*) moduli were determined by the Hill estimate. 

### 3.5. Raman, IR, and Phonon Spectra

**Figure 7:**
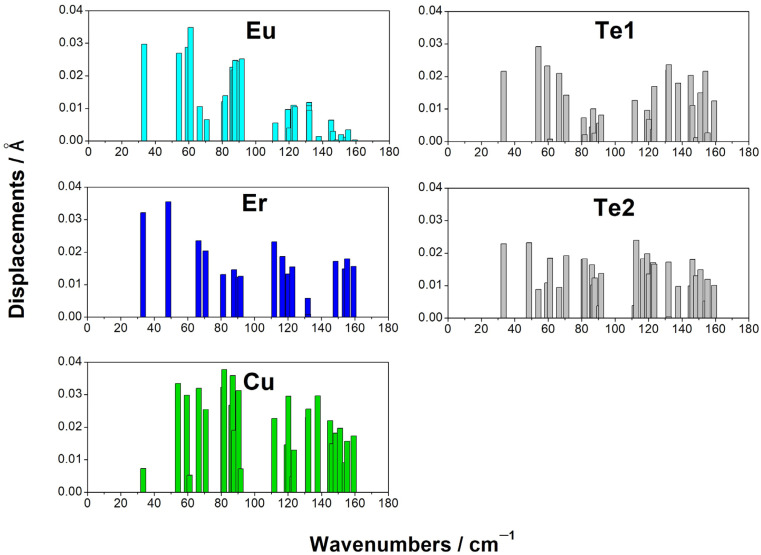
The values of the ion displacements at the phonon modes in the EuErCuTe_3_ (space group: *Cmcm*).

From the DFT calculations, the wavenumbers and types of modes were determined (Table 6). From the calculations, displacement vectors were obtained. This made it possible to evaluate the participation of each ion in a particular mode. The values of the ion displacements characterized their participation in the modes (Figure 7).

According to the calculations, the phonon spectrum of the crystal at the gamma point lied in the frequency range of up to 170 cm^−1^. In this frequency range, not only light copper ions are involved but also tellurium and erbium ions.

**Figure 8:**
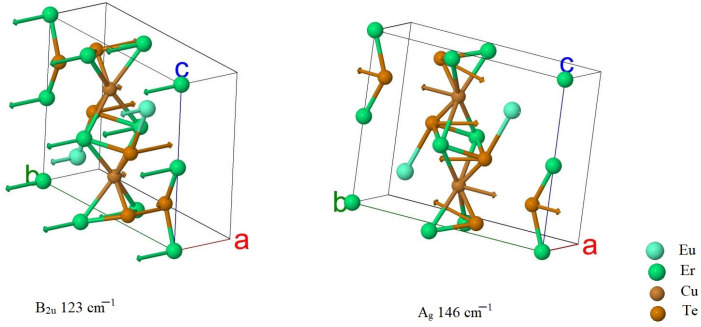
Ion displacements in the IR and Raman modes with maximum intensity.

A strong mixing of vibrations in the structural units in the crystal EuErCuSe_3_ could be noted. In crystal EuErCuTe_3,_ europium ions participate in the frequency range of up to ~95 cm^−1^. The calculations predicted a gap in the phonon spectrum in the region ~95–110 cm^−1^ (Figure 7). The calculations predicted that in the crystal EuErCuSe_3,_ the most intense Raman mode had a frequency of approximately 146 cm^−1^ (A_1g_) and the most intense infrared mode had a frequency of approximately 123 cm^−1^ (B_2u_). These modes are illustrated in Figure 8.

**Figure 9:**
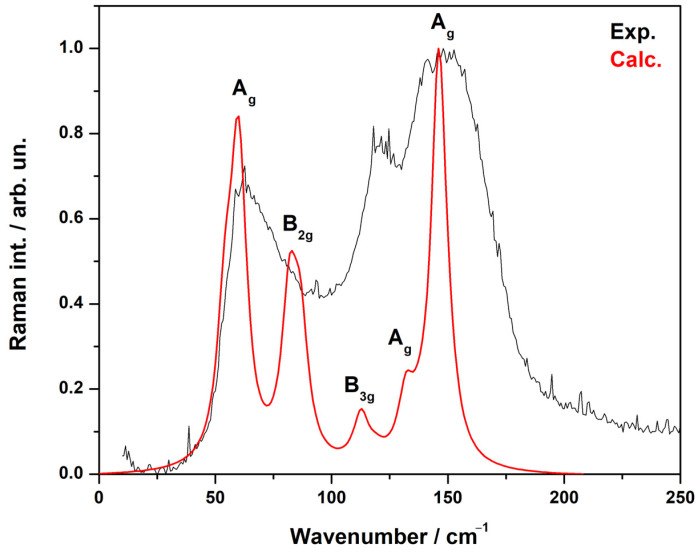
Raman spectrum modeling results. The calculations were carried out for the exciting laser wavelengths *λ* = 532 nm and *T* = 300 K.

The calculated Raman spectrum in comparison with the experimental one is shown in Figure 9. The results of calculating the phonon spectrum can be useful for interpreting IR and Raman spectra of the rare earth tellurides in Eu*Ln*CuTe_3_.

The largest ion displacement was 0.038 Å. In the case when the displacement was greater than or equal to 0.02 Å, the displacement was indicated by “S”. If the displacement did not exceed 0.01 Å, then the displacement was indicated by “W”. If the value of the displacement was less than 0.005 Å, then the ion was not mentioned in the column “Participants”.

## 4. Conclusions

This article discusses the synthesis, structure, and optical and magnetic properties of the new complex telluride EuErCuTe_3_. The compound crystallizes in the KZrCuS_3_ structure type. Its crystal structure is built from distorted [ErTe_6_]^9−^ octahedra and [CuTe_4_]^7−^ tetrahedra, forming two-dimensional layers. Trigonal prisms of [EuTe_6_]^10−^ are located between the layers. In the series of EuErCu*Ch*_3_ chalcogenides (*Ch* = S, Se, and Te), a change in the coordination polyhedron of Eu^2+^ was observed, along with a change in the structural type and space group and increases in the structural parameters. EuErCuTe_3_ contains two magnetic ions, Eu^2+^ and Er^3+^, and it undergoes a ferrimagnetic transition at 4.2 K. The obtained results correlate with the observed ferrimagnetic ordering in erbium sulfide and selenide EuErCu*Ch*_3_ (*Ch* = S and Se). EuErCuTe_3_ is paramagnetic in the temperature range 300 K to 4.2 K. Within the framework of the DFT approach, the crystal structure and the IR, Raman, and “silent” modes were studied. The elastic constants and elastic moduli were calculated. The experimental Raman spectrum of the synthesized sample was interpreted using the calculated Raman spectra of the EuErCuTe_3_. The theoretical calculations also allowed us to assign vibrational modes as well as to reveal the involved ions responsible for these modes.

## Figures and Tables

**Table 1:** Main parameters of the processing and refinement of the EuErCuTe_3_ sample.

	EuErCuTe_3_
Molecular weight	765.56
Space group	*Cmcm*
Structure type	KZrCuS_3_
*Z*	4
*a* (Å)	4.3086(3)
*b* (Å)	14.3093(9)
*c* (Å)	11.1957(7)
*V* (Å^3^)	690.25(8)
*ρ*_cal_ (g/cm^3^)	7.367
*μ* (mm^−1^)	36.369
Reflections measured	6748
Reflections independent	477
Reflections with *F*_o_ > 4*σ*(*F*_o_)	411
2*θ*_max_ (°)	27.48
*h, k, l* limits	−5 ≤ *h* ≤ 5, −18 ≤ *k* ≤ 18, −14 ≤ *l* ≤ 14
*R*_int_	0.070
*Refinement results*	
Number of refinement parameters	24
*R*_1_ with *F*_o_ > 4*σ*(*F*_o_)	0.026
w*R*_2_	0.056
*Goof*	1.084
Δ*ρ*_max_(e/Å^3^)	1.438
Δ*ρ*_min_(e/Å^3^)	−1.386
Extinction coefficient, ε	0.0026(2)
CSD-number	2261647

**Table 2:** Fractional atomic coordinates and isotropic or equivalent isotropic displacement parameters of the EuErCuTe_3_ sample.

Atom	*x/a*	*y/b*	*z/c*	*U_eq_* (Å^2^)
Eu	0	0.75485(6)	^1^/_4_	0.0288(3)
Er	0	0	0	0.0229(2)
Cu	0	0.47077(14)	^1^/_4_	0.0287(4)
Te1	0	0.08068(6)	^1^/_4_	0.0204(3)
Te2	0	0.35822(5)	0.06307(6)	0.0218(2)

**Table 3:** Atomic displacement parameters (Å^2^) of the EuErCuTe_3_ sample.

	*U*_11_	*U*_22_	*U*_33_	*U*_12_	*U*_13_	*U*_23_
Eu	0.0153(4)	0.0231(5)	0.0480(5)	0	0	0
Er	0.0155(3)	0.0205 (4)	0.0328(4)	0	0	−0.0041(3)
Cu	0.0261(10)	0.0263(10)	0.0337(10)	0	0	0
Te1	0.0166(5)	0.0177(5)	0.0269(5)	0	0	0
Te2	0.0149(3)	0.0185(4)	0.0320(4)	0	0	−0.0028(3)

**Table 4:** Bond lengths (*d*/Å) and bond angles (∠/°) in the crystal structures of the EuErCuTe_3_.

**Bond lengths**					
Eu–Te1 ^i^	3.294(1)	Er–Te1	3.0277(4)	Cu–Te2	2.641(1)
Eu–Te1 ^ii^	3.294(1)	Er–Te1 ^v^	3.0277(4)	Cu–Te2 ^x^	2.641(1)
Eu–Te2 ^iii^	3.3479(6)	Er–Te2 ^vi^	3.0423(5)	Cu–Te1 ^ii^	2.667(1)
Eu–Te2 ^i^	3.3479(6)	Er–Te2 ^vii^	3.0423(5)	Cu–Te1 ^i^	2.667(1)
Eu–Te2 ^ii^	3.3479(6)	Er–Te2 ^viii^	3.0423(5)		
**Bond angles**					
Te1 ^i^–Eu–Te1 ^ii^	81.68(3)	Te1–Er–Te1 ^v^	180.0	Te2–Cu–Te2 ^x^	104.84(7)
Te1 ^i^–Eu–Te2 ^iii^	139.03(2)	Te1–Er–Te2 ^vi^	87.72(2)	Te2–Cu–Te1 ^ii^	111.08(1)
Te1 ^ii^–Eu–Te2 ^iii^	85.03(1)	Te1 ^v^–Er–Te2 ^vi^	92.28(2)	Te2 ^x^–Cu–Te1 ^ii^	111.08(1)
Te1 ^i^–Eu–Te2 ^i^	85.03(1)	Te1–Er–Te2 ^vii^	92.28(2)	Te2–Cu–Te1 ^i^	111.08(1)
Te1 ^ii^–Eu–Te2 ^i^	139.03(2)	Te1 ^v^–Er–Te2 ^vii^	87.72(2)	Te2 ^x^–Cu–Te1 ^i^	111.08(1)
Te2 ^iii^–Eu–Te2 ^i^	127.56(3)	Te2 ^vi^–Er–Te2 ^vii^	180.00(2)	Te1 ^ii^–Cu–Te1 ^i^	107.74(8)
Te1 ^i^–Eu–Te2 ^ii^	139.03(2)	Te1–Er–Te2 ^viii^	92.28(2)		
Te1 ^ii^–Eu–Te2 ^ii^	85.03(1)	Te1 ^v^–Er–Te2 ^viii^	87.72(2)		
Te2 ^iii^–Eu–Te2 ^ii^	77.38(2)	Te2 ^vi^–Er–Te2 ^viii^	89.84(2)		
Te2 ^i^–Eu–Te2 ^ii^	80.10(2)	Te2 ^vii^–Er–Te2 ^viii^	90.16(2)		
Te1 ^i^–Eu–Te2 ^iv^	85.03(1)	Te1–Er–Te2 ^ix^	87.72(2)		
Te1 ^ii^–Eu–Te2 ^iv^	139.03(2)	Te1 ^v^–Er–Te2 ^ix^	92.28(2)		
Te2 ^iii^–Eu–Te2 ^iv^	80.10(2)	Te2 ^vi^–Er–Te2 ^ix^	90.16(2)		
Te2 ^i^–Eu–Te2 ^iv^	77.38(2)	Te2 ^vii^–Er–Te2 ^ix^	89.84(2)		
Te2 ^ii^–Eu–Te2 ^iv^	127.56(3)	Te2 ^viii^–Er–Te2 ^ix^	180.00(2)		

Symmetry codes: (i) x − ^1^/_2_, y+^1^/_2_, z; (ii) x+^1^/_2_, y+^1^/_2_, z; (iii) x+^1^/_2_, y+^1^/_2_, −z+^1^/_2_; (iv) x − ^1^/_2_, y+^1^/_2_, −z+^1^/_2_; (v) −x, −y, −z; (vi) −x+^1^/_2_, −y+^1^/_2_, −z; (vii) x − ^1^/_2_, y − ^1^/_2_, z; and (viii) x+^1^/_2_, y − ^1^/_2_, z; (ix) −x − ^1^/_2_, −y+^1^/_2_, −z; (x) x, y, −z+^1^/_2_.

**Table 5:** The elastic constants and modulus, and also the Vickers hardness (GPa), of EuErCuTe_3_.

C_11_	C_12_	C_13_	C_22_	C_23_	C_33_	C_44_	C_55_	C_66_	Averaging Scheme	*B*	*G*	*Y*	Poisson’s Ratio	*H_V_*
122	52	40	95	47	113	10	31	45	Voigt	68	30	78	0.307	3.2
									Reuss	67	23	61	0.349	
									Hill	67	26	70	0.328	

**Table 6:** Phonons at the gamma point of the EuErCuTe_3_.

Frequency, cm^−1^	Type	IR		Raman		Involved Ions ^1^
		Active/ Inactive	Intensity IR (km·mol^−1^)	Active/ Inactive	Intensity Raman (Arbitrary Units)	
**33**	B_1u_	A	10	I		Eu ^S^, Er ^S^, Cu ^W^, Te1 ^S^, Te2 ^S^
48	A_u_	I	0	I		Er ^S^, Te2 ^S^
54	B_1g_	I	0	A	358	Eu ^S^, Cu ^S^, Te1 ^S^, Te2 ^W^
59	A_g_	I	0	A	582	Eu ^S^, Cu ^S^, Te1 ^S^, Te2
61	B_2g_	I	0	A	287	Eu ^S^, Cu ^W^, Te2
67	B_2u_	A	18	I		Eu, Er ^S^, Cu ^S^, Te1 ^S^, Te2 ^W^
71	B_1u_	A	14	I		Eu ^W^, Er ^S^, Cu ^S^, Te1, Te2
81	B_3u_	A	49	I		Eu, Er, Cu ^S^, Te1 ^W^, Te2
82	B_2g_	I	0	A	415	Eu, Cu ^S^, Te2
86	A_g_	I	0	A	136	Eu ^S^, Cu ^S^, Te2
87	B_1g_	I	0	A	202	Eu ^S^, Cu ^S^, Te1, Te2
88	B_1u_	A	90	I		Eu ^S^, Er, Cu, Te2
90	B_2u_	A	2	I		Eu ^S^, Er, Cu ^S^, Te1 ^W^
92	B_3u_	A	115	I		Eu ^S^, Er, Cu ^W^, Te1 ^W^, Te2
112	B_3u_	A	16	I		Eu ^W^, Er ^S^, Cu ^S^, Te1
113	B_3g_	I	0	A	132	Te2 ^S^
117	A_u_	I	0	I		Er, Te2
119	B_1g_	I	0	A	25	Eu ^W^, Cu, Te1 ^W^, Te2
120	B_1u_	A	374	I		Er, Cu ^S^, Te1 ^W^, Te2
122.66	B_2u_	A	495	I		Eu, Er, Te2
123.50	B_2g_	I	0	A	1.5	Eu, Cu, Te1, Te2
131.92	B_3u_	A	79	I		Eu, Er ^W^, Cu, Te1 ^S^, Te2
131.95	B_1g_	I	0	A	47	Eu, Cu ^S^, Te1 ^S^, Te2 ^W^
131.97	A_g_	I	0	A	119	Eu, Te1, Te2
132.07	B_2u_	A	135	I		Eu ^W^, Cu ^S^, Te1 ^S^
138	A_g_	I	0	A	51	Cu ^S^, Te1, Te2 ^W^
145	B_2g_	I	0	A	116	Eu ^W^, Cu ^S^, Te1 ^S^, Te2 ^W^
146	A_g_	I	0	A	1000	Cu, Te1, Te2
148	B_1u_	A	5	I		Er, Cu, Te2
151	B_2g_	I	0	A	59	Cu, Te1, Te2
154	B_1u_	A	137	I		Er, Cu ^W^, Te1 ^S^, Te2 ^W^
155	B_3u_	A	185	I		Er, Cu, Te2
159	B_3u_	A	2	I		Er, Cu, Te1, Te2

^1^ The superscripts “S” and “W” denote strong and weak ion displacements in the modes, respectively.

## Data Availability

Data are available from the authors on request.

## References

[1] Duczmal M., Pawlak L. (1994). Magnetic properties of Tl*Ln*S_2_ compounds (*Ln* = Nd, Gd, Dy, Er and Yb). J. Alloys Compd..

[2] Ahmed N., Nisar J., Kouser R., Nabi A.G., Mukhtar S., Saeed Y., Nasim M.H. (2017). Study of electronic, magnetic and optical prop-erties of K*M*S_2_ (*M* = Nd, Ho, Er and Lu): First principle calculations. Mater. Res. Expr..

[3] Azarapin N.O. (2022). Synthesis, Structure and Properties of Compounds Ba*RE*CuS_3_ (*RE* = Rare Earth Element). Ph.D. Thesis.

[4] Esmaeili M., Forbes S., Tseng Y.C., Mozharivskyj Y. (2014). Crystal Structure, Electronic and Physical Properties of Monoclinic *RE*CuTe_2_ in Contrast to *RE*CuSe_2_ (*RE*: Pr, Sm, Gd, Dy and Er). Solid State Sci..

[5] Esmaeili M., Tseng Y.-C., Mozharivskyj Y. (2014). Thermoelectric properties, crystal and electronic structure of semiconducting *RE*CuSe_2_ (*RE* = Pr, Sm, Gd, Dy and Er). J. Alloys Compd..

[6] Yao J., Deng B., Sherry L.J., McFarland A.D., Ellis D.E., van Duyne R.P., Ibers J.A. (2004). Syntheses, structure, some band gaps, and electronic structures of Cs*Ln*ZnTe_3_ (*Ln* = La, Pr, Nd, Sm, Gd, Tb, Dy, Ho, Er, Tm, Y). Inorg. Chem..

[7] Mitchell K., Huang F.Q., Caspi E.N., McFarland A.D., Haynes C.L., Somers R.C., Jorgensen J.D., Van Duyne R.P., Ibers J.A. (2004). Syntheses, structure, and selected physical properties of Cs*Ln*MnSe_3_ (*Ln* = Sm, Gd, Tb, Dy, Ho, Er, Tm, Yb, Y) and *A*YbZn*Q*_3_ (*A* = Rb, Cs; *Q* = S, Se, Te). Inorg. Chem..

[8] Yin W., Wang W., Bai L., Feng K., Shi Y., Hao W., Yao J., Wu Y. (2012). Syntheses, structures, physical properties, and electronic structures of Ba_2_*MLn*Te_5_ (*M* = Ga and *Ln* = Sm, Gd, Dy, Er, Y; *M* = In and *Ln* = Ce, Nd, Sm, Gd, Dy, Er, Y). Inorg. Chem..

[9] Yin W., Feng K., Wang W., Shi Y., Hao W., Yao J., Wu Y. (2012). Syntheses, structures, optical and magnetic properties of Ba_2_*MLn*Se_5_ (*M* = Ga, In; *Ln* = Y, Nd, Sm, Gd, Dy, Er). Inorg. Chem..

[10] Ruseikina A.V., Andreev O.V., Galenko E.O., Koltsov S.I. (2017). Trends in thermodynamic parameters of phase transitions of lan-thanide sulfides Sr*Ln*CuS_3_ (*Ln* = La–Lu). J. Therm. Anal. Calorim..

[11] Ruseikina A.V., Solovyov L.A. (2019). Grigoriev, M.V.; Andreev, O.V. Crystal structure variations in the series Sr*Ln*CuS_3_ (*Ln* = La, Pr, Sm, Gd, Er and Lu). Acta Crystallogr..

[12] Ruseikina A.V., Soloviev L.A., Galenko E.O., Grigoriev M.V. (2018). Refined Crystal Structures of Sr*Ln*CuS_3_ (*Ln* = Er, Yb). Russ. J. Inorg. Chem..

[13] Huang F.Q., Ibers J.A. (2001). Syntheses and structures of the quaternary copper tellurides K_3_*Ln*_4_Cu_5_Te_10_ (*Ln* = Sm, Gd, Er), Rb_3_*Ln*_4_Cu_5_Te_10_ (*Ln* = Nd, Gd), and Cs_3_Gd_4_Cu_5_Te_10_. J. Solid State Chem..

[14] Ikhioya I.L., Nkele A.C., Chigozirim E.M., Aisida S.O., Maaza M., Ezema F.I. (2020). Effects of Erbium on the Properties of Electro-chemically-Deposited Zirconium Telluride Thin Films. Nanoarchitectonics.

[15] Ruseikina A.V., Chernyshev V.A., Velikanov D.A., Aleksandrovsky A.S., Shestakov N.P., Molokeev M.S., Grigoriev M.V., Andreev O.V., Garmonov A.A., Matigorov A.V. (2021). Regularities of the property changes in the compounds Eu*Ln*CuS_3_ (*Ln* = La–Lu). J. Alloys Compd..

[16] Andreev O.V., Atuchin V.V., Aleksandrovsky A.S., Denisenko Y.G., Zakharov B.A., Tyutyunnik A.P., Habibullayev N.N., Velikanov D.A., Ulybin D.A., Shpindyuk D.D. (2022). Synthesis, structure, and properties of Eu*Ln*CuSe_3_ (*Ln* = Nd, Sm, Gd, Er). Crystals.

[17] Koscielski L.A., Ibers J.A. (2012). The Structural Chemistry of Quaternary Chalcogenides of the Type *AMM’Q*_3_. Z. Anorg. Allg. Chem..

[18] Ruseikina A.V., Solovyov L.A., Chernyshev V.A., Aleksandrovsky A.S., Andreev O.V., Krylova S.N., Krylov A.S., Velikanov D.A., Molokeev M.S., Maximov N.G. (2019). Synthesis, structure, and properties of EuErCuS_3_. J. Alloys Compd..

[19] Boncher W., Dalafu H., Rosa N., Stoll S. (2015). Europium chalcogenide magnetic semiconductor nanostructures. Coord. Chem. Rev..

[20] Wolf M., Sürgers C., Fischer G., Scherer T., Beckmann D. (2014). Fabrication and magnetic characterization of nanometer-sized ellipses of the ferromagnetic insulator EuS. J. Magn. Magn. Mater..

[21] He W., Somarajan S., Koktysh D.S., Dickerson J.H. (2010). Superantiferromagnetic EuTe nanoparticles: Room temperature colloidal synthesis, structural characterization, and magnetic properties. Nanoscale.

[22] Oliveira N.F., Foner S., Shapira Y., Reed T.B. (1972). EuTe. I. Magnetic Behavior of Insulating and Conducting Single Crystals. Phys. Rev..

[23] Chen J., Dresselhaus G., Dresselhaus M., Springholz G., Bauer G. (1994). Magnetic Properties of Heisenberg Antiferromagnetic EuTe/PbTe Superlattices. MRS Proc..

[24] Kępa H., Springholz G., Giebultowicz T.M., Goldman K.I., Majkrzak C.F., Kacman P., Blinowski J., Holl S., Krenn H., Bauer G. (2003). Magnetic interactions in EuTe epitaxial layers and EuTe/PbTe superlattices. Phys. Rev. B.

[25] Li Y., Liu J., Zhang P., Jing Q., Liu X., Zhang J., Xiao N., Yu L., Niu P. (2021). Electrical transport properties of EuTe under high pressure. J. Mater. Chem. C.

[26] Shannon R.D. (1976). Revised effective ionic radii and systematic studies of interatomic distances in halides and chalcogenides. Acta Crystallogr..

[27] Pal K., Hua X., Xia Y., Wolverton C. (2019). Unraveling the Structure-Valence-Property Relationships in *AMM′Q*_3_ Chalcogenides with Promising Thermoelectric Performance. ACS Appl. Energy Mater..

[28] Pal K., Xia Y., Shen J., He J., Luo Y., Kanatzidis M.G., Wolverton C. (2021). Accelerated discovery of a large family of quaternary chalcogenides with very low lattice thermal conductivity. npj Comput. Mater..

[29] Ruseikina A.V., Andreev O.V., Demchuk Z.A. (2016). Preparation of polycrystalline samples of the Eu*Ln*CuS_3_ (*Ln* = Gd, Lu) compounds. Inorg. Mater..

[30] Solovyeva A.V. (2012). Regularities of phase equilibria in the systems *A*^II^S–FeS, *A*^II^S–FeS–*Ln*_2_S_3_, *A*^II^S–Cu_2_S–*Ln*_2_S_3_ (*A*^II^ = Mg, Ca, Sr, Ba; *Ln* = La–Lu). Ph.D. Thesis.

[31] Sikerina N.V. (2005). Regularities of Phase Equilibria in SrS–*Ln*_2_S_3_–Cu_2_S Systems, Preparation and Structure of Sr*Ln*CuS_3_ Compounds. Ph.D. Thesis.

[32] Wakeshima M., Furuuchi F., Hinatsu Y. (2004). Crystal structures and magnetic properties of novel rare-earth copper sulfides, Eu*R*CuS_3_ (*R* = Y, Gd–Lu). J. Phys. Condens. Matter.

[33] Rare-Earth Metal Long Term Air Exposure Test, Metallium, Inc. https://www.elementsales.com/re_exp/.

[34] Keil P., Lutzenkirchen-Hecht D., Frahm R. (2007). Investigation of room temperature oxidation of Cu in Air by YonedaXAFS. AIP Conf. Proc..

[35] Sheldrick G.M. (2008). A short history of SHELX. Acta Crystallogr..

[36] Spek (2008). A.L. PLATON—A Multipurpose Crystallographic Tool.

[37] Crystal. http://www.crystal.unito.it/index.php.

[38] Energy-Consistent Pseudopotentials of the Stuttgart/Cologne Group. http://www.tc.uni-koeln.de/PP/clickpse.en.html.

[39] Towler M. CRYSTAL Resourses Page. https://vallico.net/mike_towler/crystal.html.

[40] Grigoriev M.V., Solovyov L.A., Ruseikina A.V., Aleksandrovsky A.S., Chernyshev V.A., Velikanov D.A., Garmonov A.A., Molokeev M.S., Oreshonkov A.S., Shestakov N.P. (2022). Quaternary Selenides Eu*Ln*CuSe_3_: Synthesis, Structures, Properties and In Silico Studies. Int. J. Mol. Sci..

[41] Yang L., Powell D.R., Houser R.P. (2007). Structural variation in copper(i) complexes with pyridylmethylamide ligands: Structural analysis with a new four-coordinate geometry index, τ_4_. Dalton Trans..

[42] Ruseikina A.V., Grigoriev M.V., Garmonov A.A., Molokeev M.S., Schleid T., Safin D.A. (2023). Synthesis, structures and magnetic properties of the Eu-based quaternary tellurides EuGdCuTe_3_ and EuLuCuTe_3_. Cryst. Eng. Comm..

[43] Ruseikina A.V., Grigoriev M.V., Solovyov L.A., Molokeev M.S., Garmonov A.A., Velikanov D.A., Safin D.A. (2023). Unravelling the rare-earth (*RE*) element-induced magnetic and optical properties in the structures of quaternary selenides Sr*RE*CuSe_3_. Inorg. Chem. Commun..

[44] Pavlyuk M.D. (2020). Detector Crystals Based on CdTe and Cd_1–*x*_Zn*_x_*Te for Direct Detection of X-ray and Gamma Quanta. Ph.D. Thesis.

[45] Ruseikina A.V., Molokeev M.S., Chernyshev V.A., Aleksandrovsky A.S., Krylov A.S., Krylova S.N., Velikanov D.A., Grigoriev M.V., Maximov N.G., Shestakov N.P. (2021). Synthesis, structure, and properties of EuScCuS_3_ and SrScCuS_3_. J. Solid State Chem..

[46] Wu J., Zhao E.J., Xiang H.P., Hao X.F., Liu X.J., Meng J. (2007). Crystal structures and elastic properties of superhard IrN_2_ and IrN_3_ from first principles. Phys. Rev..

[47] Korabelnikov D.V., Zhuravlev Y.N. (2016). Ab-initio investigations of the elastic properties of chlorates and perchlorates. Phys. Solid State.

[48] Tian Y., Xu B., Zhao Z. (2012). Microscopic theory of hardness and design of novel superhard crystals. Int. J. Refract. Met. Hard Mater..

